# Complete Closed Genome Sequences of *Mannheimia haemolytica* Serotypes A1 and A6, Isolated from Cattle

**DOI:** 10.1128/genomeA.00188-13

**Published:** 2013-05-16

**Authors:** Gregory P. Harhay, Sergey Koren, Adam M. Phillippy, D. Scott McVey, Jennifer Kuszak, Michael L. Clawson, Dayna M. Harhay, Michael P. Heaton, Carol G. Chitko-McKown, Timothy P. L. Smith

**Affiliations:** USDA, ARS, U.S. Meat Animal Research Center, Clay Center, Nebraska, USAa;; National Biodefense Analysis and Countermeasures Center, Frederick, Maryland, USAb;; USDA, ARS, Center for Grain and Animal Health Research, Manhattan, Kansas, USAc;; Nebraska Veterinary Diagnostic Center, School of Veterinary Medicine and Biomedical Sciences, University of Nebraska—Lincoln, Lincoln, Nebraska, USAd

## Abstract

*Mannheimia haemolytica* is a respiratory pathogen affecting cattle and related ruminants worldwide. *M. haemolytica* is commonly associated with bovine respiratory disease complex (BRDC), a polymicrobial multifactorial disease. We present the first two complete closed genome sequences of this species, determined using an automated assembly pipeline requiring no manual finishing.

## GENOME ANNOUNCEMENT

*Mannheimia haemolytica* is a Gram-negative rod bacterium commonly associated with severe acute hemorrhagic fibrinonecrotic bronchopneumonia (1, 2) in feedlot cattle, although it can be found in the nasopharynx of asymptomatic cattle. The bovine respiratory disease complex (BRDC) progression to bronchopneumonia results from host, pathogen, and environmental interactions that are not completely understood. Stress, viral infections, extreme weather changes, transportation, and other factors appear to predispose cattle to bronchopneumonia that is symptomatic of BRDC (3, 4). A typical BRDC outbreak occurs about 7 to 10 days after calves arrive at a feedlot. During this time, calves are at the highest risk for BRDC. Draft genome sequences of *M. haemolytica* serotypes A1 (5) and A2 (6, 7) have shown that *M. haemolytica* frequently harbors antibiotic resistance cassettes, mobile elements, and other determinants that enhance its virulence. Here, we report the first complete closed genomes of any *M. haemolytica* serotypes.

*M. haemolytica* strains USDA-ARS-USMARC 183 and USDA-ARS-USMARC 185 (serotypes A1 and A6, respectively) were among many *M. haemolytica* BRDC clinical case isolates that were chosen for sequencing. Genomic DNA was extracted using a Qiagen blood and cell culture DNA kit and Genomic-tip 100/G columns according to the directions of each manufacturer. Closed genomes were generated using a hybrid assembly pipeline in two steps. In step one, 50-fold coverage of Roche GS-FLX Titanium reads was used to error-correct continuous long Pacific BioScience RS sequencer reads (clPBr) (C2 chemistry) using the pacBioToCA pipeline (8). In step two, the resulting 10,000 error-corrected clPBr (25-fold coverage, minimum length of 6 kb) were assembled with the Celera assembler version 7 (9). Both genomes were spanned by a single contig. A dot plot of these contigs showed that the 5′ end of the serotype A1 genome overlapped the 3′ end by 6,021 bp with >99.8% pairwise identity, while the 5′ end of the serotype A1 genome overlapped the 3′ end by 3,144 bp with 99.9% pairwise identity. For each contig of each serotype, the overlapping region was deleted from the 3′ end and the two ends were joined to circularize the chromosome. GenSkew (http://genskew.csb.univie.ac.at) was used to localize the approximate origin of replication of the chromosome so that the base pair numbering could be reindexed to reflect the origin of replication at base pair position 1. A local instance of Do-It-Yourself Annotator (DIYA) (10) was used to annotate the circularized chromosome. The serotype A6 genome has 2,544,668 bp, a G+C content of 40.83%, 2,537 coding sequences (CDSs), 20 rRNAs, and 60 tRNAs, while the serotype A1 genome has 2,658,332 bp, a G+C content of 40.91%, 2,731 CDSs, 20 rRNAs, and 64 tRNAs.

### Nucleotide sequence accession numbers.

The completed genome sequences of *M. haemolytica* USDA-ARS-USMARC 183 and USDA-ARS-USMARC 185 were deposited in GenBank under accession no. CP004752 and CP004753, respectively.
